# Pathogenic Role of Iron Deposition in Reticuloendothelial Cells during the Development of Chronic Hepatitis C

**DOI:** 10.1155/2013/686420

**Published:** 2013-04-04

**Authors:** Hironori Mitsuyoshi, Kohichiroh Yasui, Kanji Yamaguchi, Masahito Minami, Takeshi Okanoue, Yoshito Itoh

**Affiliations:** ^1^Molecular Gastroenterology and Hepatology, Graduate School of Medical Science, Kyoto Prefectural University of Medicine, Kawaramachi Hirokouji, Kamigyo-ku, Kyoto 602-8566, Osaka 564-0013, Japan; ^2^Saiseikai Suita Hospital, Suita, Japan

## Abstract

*Aim*. Chronic hepatitis C (CHepC) is frequently associated with hepatic iron overload, yet mechanisms underlying iron-induced liver injury have not been elucidated. We examined the significance of iron deposition in hepatocytes (HC) and reticuloendothelial cells (REC) in CHepC. *Methods*. Stainable hepatic iron was scored according to the iron deposition pattern in 373 patients. The levels of serum soluble TNF-**α** receptor (sTNFR2) and hepatic hepcidin mRNA and the efficacy of phlebotomy were compared among patients with different iron deposition patterns. *Results*. Serum transaminase levels and hepatic scores of stage, grade, and steatosis were higher in patients with REC iron staining than in those without. REC iron scores were independently associated with advanced stage. Serum sTNFR2 levels were significantly higher in patients with REC iron than in those without. REC iron scores were independently correlated with sTNFR2 levels. Compared with patients without stainable iron, those with iron overload had decreased ratios of hepcidin mRNA to serum ferritin. The efficacy of phlebotomy was greater in patients with REC iron than in those without REC iron. *Conclusions*. The present results show the importance of REC iron for the development of CHepC and the therapeutic effect of phlebotomy in CHepC.

## 1. Introduction

Chronic hepatitis C (CHepC) is frequently associated with hepatic iron overload [1–3]. Elevation of serum iron indices or stainable hepatic iron has been shown in 40 to 70% of patients with CHepC [1–3]. From these observations, iron-induced oxidative stress has been considered to be an underlying mechanism of liver injury and of development of hepatocellular carcinoma [4–6].

The mechanisms of hepatic iron overload in CHepC have not yet been elucidated. However, hepcidin has attracted much attention as an important factor in the disease process. Hepcidin is exclusively produced in the liver and regulates body iron stores [7, 8]. Hepcidin causes internalization and degradation of iron-transporter ferroportin on duodenal enterocytes and macrophages, thereby blocking iron absorption and iron recycling, respectively [9]. In hereditary hemochromatosis (HH), defective hepcidin synthesis results in a subsequent increase in body iron stores [10]. In CHepC, hepatic iron overload has been attributed to the mutation of the hemochromatosis protein (HFE) gene [11], since several reports have found an association between HFE genotypes and iron overload in patients with CHepC [12–14]. Another possible mechanism is the direct effect of the hepatitis C virus (HCV) on hepcidin synthesis [15]. Transgenic mice expressing HCV polyprotein have been shown to have decreased hepatic expression of hepcidin due to HCV-induced oxidative stress [15].

When hepatic iron overload develops, stainable iron can be seen either in hepatocytes (HC), reticuloendothelial cells (REC), or both cell types [16]. Recently, patterns of hepatic iron distribution have attracted a considerable attention in chronic liver diseases, since the patterns would predict the histological progressions. In particular, nonparenchymal iron deposition has been associated with advanced stages of alcoholic liver disease (ALD) and nonalcoholic steatohepatitis (NASH) [17, 18]. In CHepC, Di Bisceglie et al. initially reported the presence of hepatic iron deposition both in HC and REC [19]. Hézode et al. reported the positive relationship between histological activity and iron deposition either in REC or mixed HC/REC in patients with CHepC [2].

The mechanism and pathogenicity underlying hepatic iron distribution still remain unclear. However, hepcidin is one of the candidates that could potentially resolve these issues. Hepcidin is synthesized by HC in response to iron overload [7] and can sequestrate iron in Kupffer cells and macrophages through the downregulation of ferroportin [9]. Thus, hepcidin can modify outcomes of patients with CHepC by determining iron deposition patterns.

In the present study, relationships between iron deposition patterns and histological scores in CHepC were examined. Then, levels of TNF-*α* and hepatic hepcidin mRNA and the effect of phlebotomy on liver function tests were compared among patients with different iron deposition patterns. The present study examines the significance of nonparenchymal iron deposition and discusses the mechanisms and pathogenicity underlying iron deposition patterns.

## 2. Patients and Methods

### 2.1. Patients

Patients with CHepC who underwent liver biopsies at our institutes between January 2007 and April 2012 were retrospectively reviewed. Patients were selected according to the following criteria: positive anti-HCV antibody; positive serum HCV-RNA confirmed by reverse transcription-polymerase chain reaction (RT-PCR); no history of antiviral therapy; no excessive alcohol intake (intake less than 40 g/week); negative for hepatitis B surface antigen or antibodies to human immunodeficiency virus; and absence of other forms of chronic liver disease, including autoimmune hepatitis, primary biliary cirrhosis, and primary sclerosing cholangitis. Anthropometry and laboratory data were collected from all patients at the time of the liver biopsy. Informed written consent was obtained from each patient. The study protocol conformed to the ethical guidelines of the Declaration of Helsinki approved by the Ethics Committee of the Kyoto Prefectural University of Medicine.

### 2.2. Laboratory Determination

After a 12 h overnight fast, venous blood samples were drawn to determine aspartate aminotransferase (AST), alanine aminotransferase (ALT), fasting plasma glucose (FPG), immunoreactive insulin (IRI), total cholesterol, triglycerides, and ferritin levels. The index of insulin resistance was calculated only in patients without overt diabetes (FPG >126 mg/dL), according to the homeostasis model assessment (HOMA). The formula used for insulin resistance was as follows: HOMA-R = FPG (mg/dL) × IRI (*μ*U/mL)/405.

HCV-RNA levels were determined by RT-PCR. HCV genotypes were determined by PCR of the core region with genotype-specific PCR primers [20]. HCV serogroups 1 and 2 were determined by a serologic genotyping assay [21].

Serum TNF-*α* concentrations were evaluated by the soluble TNF-*α* receptor type 2 (sTNFR2) levels, since sTNFR2 levels can be easily and stably measured and have been shown to be associated with the serum level of TNF-*α* [22]. For measurement of sTNFR2 concentrations, serum was stored at −80°C until use. The serum sTNFR2 levels were then measured in 148 patients using a commercial, sensitive enzyme-linked immunosorbent assay kit (R&D Systems, Minneapolis, MN, USA).

### 2.3. Histological Evaluation

Formalin-fixed and paraffin-embedded liver biopsy specimens were stained with hematoxylin-eosin, Masson's trichrome, and Berlin blue. Histopathological diagnosis was based on the scoring of the New Inuyama Classification [23]. Briefly, degree of hepatic fibrosis (stage) was scored as follows: 0 = none, 1 = portal expansion, 2 = bridging fibrosis, 3 = bridging fibrosis with lobular distortion, and 4 = cirrhosis. Degree of inflammation (grade) was scored as follows: 0 = none, 1 = mild, 2 = moderate, and 3 = severe. Steatosis was assessed according to the percentage of hepatocytes containing fat droplets: 0 = less than 5%, 1 = 5–9%, 2 = 10–29%, and 3 = more than 29%. HC iron deposition was scored from 0 to 4 as described previously [24]. REC iron deposition was scored from 0 to 2: 0 = scarcely seen, 1 = sporadically seen in the acinar and/or the portal tract, and 3 = frequently seen in the acinar and/or the portal tract. We considered cellular iron deposition only when granular iron deposition was observed.

### 2.4. Quantification of Hepatic mRNA Levels of Hepcidin

Hepatic mRNA levels of hepcidin were measured in 84 patients whose biopsy specimens were available. Total RNA was isolated using the TRIzol Reagent (Life Technologies, Carlsbad, CA, USA). The PCR mixture contained first-strand cDNA and specific primers for human hepcidin: sense, 5′-ACCAGAGCAAGCTCAAGACC-3′ and antisense, 5′-AAACAGAGCCACTGGTCAGG-3′. Real-time PCR was performed to quantify mRNA levels of the target genes using the StepOnePlus Real-Time PCR system (Life Technologies), and mRNA levels of hepcidin were normalized to those of *β*-actin: sense, 5′-CTGGAACGGTGAAGGTGACA-3′ and antisense, 5′-AAGGGACTTCCTGTAACAATGCA-3′.

### 2.5. Phlebotomy

Phlebotomy was received in 48 patients after the liver biopsy. All patients showed elevated serum ferritin levels and/or persistent abnormal ALT levels, and none showed anemia (hemoglobin <11.0 g/dL). They underwent phlebotomy (200–400 mL) either biweekly or monthly until serum ferritin levels were <20 ng/mL. However, treatments were terminated irrespective of serum ferritin levels when blood hemoglobin concentrations decreased to less than 10 g/dL.

### 2.6. Statistical Analysis

Differences and correlations between quantitative variables were analyzed using the Student's *t*-test and the Pearson product-moment correlation coefficient, respectively. Distributions of qualitative variables were compared using the Chi-squared test. When differences between variables were considered among more than two groups, post hoc comparisons (*Bonferroni test*) were employed after the analysis of variance (ANOVA). Logistic regression model was used to analyze independent variables associated with advanced fibrosis. Multiple-regression model was used to analyze independent variables associated with sTNFR2 levels. A *P* value of less than 0.05 was considered significant.

## 3. Results

### 3.1. General Characteristics of Patients

Three hundred and seventy-three patients met the eligibility criteria. Overall, 208 patients (56%) had HC iron deposition and 125 patients (34%) had REC iron deposition, comprising of no stainable iron in 141 patients (None group), HC deposition alone in 107 (HC group), mixed HC/REC deposition in 101 (Mix group), and REC deposition alone in 24 patients (REC group). Receiver operating characteristic analysis showed that the cut-off values of ferritin for iron deposition in HC and REC were 104.5 ng/mL (area under the curve: AUC = 0.832, *P* < 0.0000001) and 224.5 ng/mL (AUC = 0.827, *P* < 0.0000001), respectively. The differences among the 4 groups were significant with regards to gender, body mass index (BMI), and serum levels of AST, ALT, and ferritin (Table 1). There were a greater number of male patients in the Mix and REC groups than in the other groups. Serum levels of AST and ALT were significantly higher in the Mix and REC groups than in the other groups. Ferritin levels were significantly higher in the Mix group than in the other groups and were significantly higher in the HC and REC groups than in the None group (Table 1).

### 3.2. Results of Liver Biopsies

The results of liver biopsies are summarized in Table 1. Iron deposition patterns were significantly associated with stage, grade, and steatosis. HC iron scores were significantly higher in the Mix group than in the HC group (*P* < 0.0005, Chi-squared test). Patients in the Mix and REC groups had higher scores of stage, grade, and steatosis than those of the other groups. In contrast, patients in the HC group had similar scores of stage, grade, and steatosis compared to patients in the None group.

### 3.3. Association between REC Iron Deposition and Fibrosis

In order to examine the variables associated with the fibrosis, patients with an early fibrosis stage were compared with those with an advanced fibrosis stage (Table 2). Age, BMI, levels of AST, ALT, FPG, IRI, and ferritin, HOMA-R, and hepatic scores of grade, steatosis, and REC iron were significantly higher in patients with advanced stage than in those with early stage. The presence of diabetes was significantly associated with advanced stage. On logistic regression analysis, serum levels of AST, ALT, and ferritin and hepatic scores of grade and REC iron were independently associated with advanced stage.

### 3.4. Association between REC Iron Scores and Serum sTNFR2 Levels

**Figure 1:**
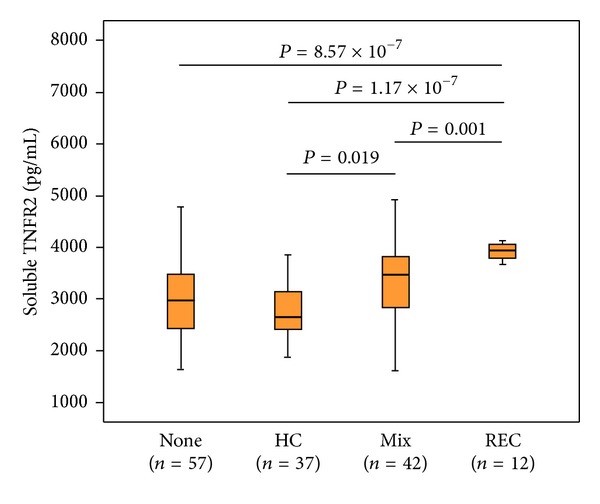
Distributions of serum soluble TNF-*α* (sTNFR2) levels among patients with different iron deposition patterns are seen. None: no stainable iron, HC: iron deposition in hepatocytes alone, Mix: iron deposition in mixed hepatocytes/reticuloendothelial cells, and REC: iron deposition in reticuloendothelial cells alone. Differences between the groups were analyzed by post hoc comparisons (Bonferroni test).

Serum sTNFR2 levels were measured in 148 patients.  Figure 1 represents the distributions of sTNFR2 levels among patients with different iron deposition patterns. Serum sTNFR2 levels were significantly higher in the REC group than in the other groups and were significantly higher in the Mix group than in the HC group. Serum sTNFR2 levels were significantly correlated with age, serum levels of AST, ALT, and ferritin, and hepatic scores of stage, grade, steatosis, and REC iron (Table 3). On regression analysis, age and hepatic scores of grade and REC iron were independently correlated with sTNFR2 levels (Table 3).

### 3.5. Hepcidin mRNA Levels

**Figure 2:**
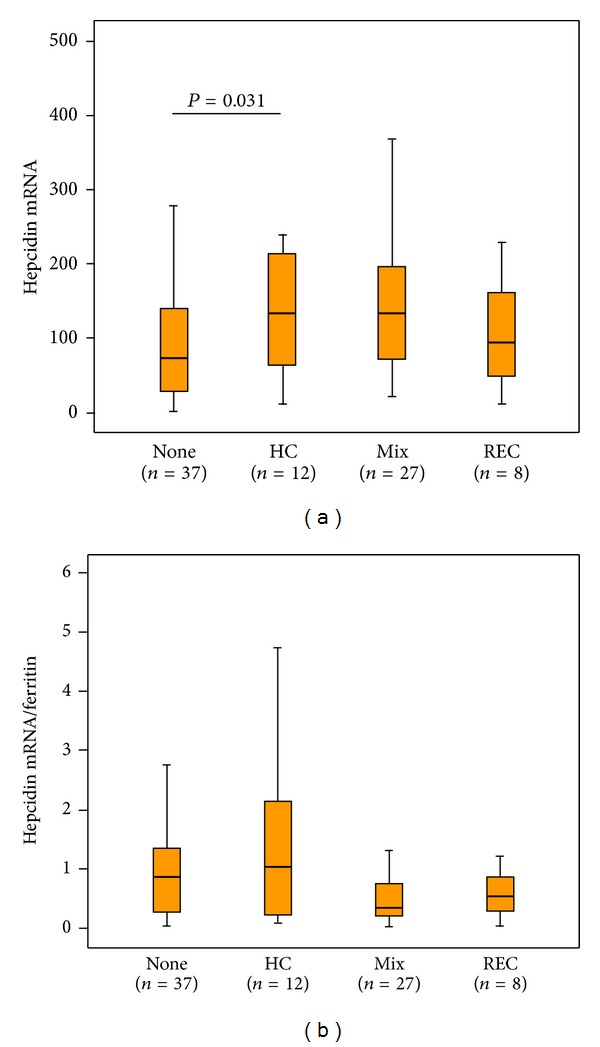
Distributions of absolute hepatic hepcidin mRNA levels (a) and hepcidin mRNA levels normalized to serum ferritin concentrations (b) among patients with different iron deposition patterns. mRNA levels of hepcidin were normalized to those of *β*-actin. None: no stainable iron, HC: iron deposition in hepatocytes alone, Mix: iron deposition in mixed hepatocytes/reticuloendothelial cells, and REC: iron deposition in reticuloendothelial cells alone. Differences between the None and HC groups were analyzed by post hoc comparisons (Bonferroni test).

Hepcidin mRNA levels were quantified in 84 patients. Overall, hepatic hepcidin mRNA levels were higher in patients with stainable iron than in those without stainable iron and the difference was achieved significance between the None and HC groups (Figure 2(a)). However, this significance disappeared after normalization relative to ferritin concentrations (Figure 2(b)). These corrected values tended to be lower in the Mix and REC groups than in the None group (Figure 2(b)).

### 3.6. Efficacy of Phlebotomy on ALT Levels

**Figure 3:**
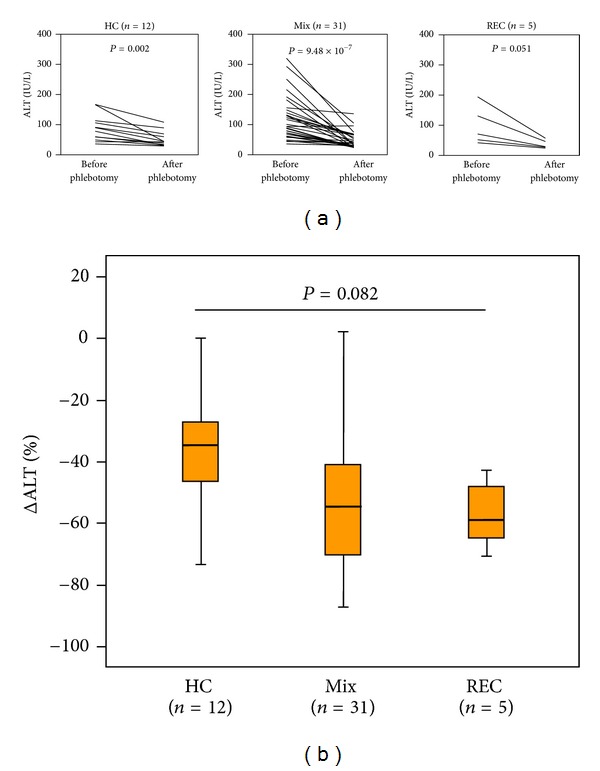
Line graphs (a) show the alanine aminotransferase (ALT) levels before and after phlebotomy. Differences in ALT levels were analyzed by the Wilcoxon signed-rank test. Box graph (b) shows the percentage change in ALT levels after phlebotomy. Differences among the three groups were analyzed by ANOVA (*P* = 0.082). None: no stainable iron, HC: iron deposition in hepatocytes alone, Mix: iron deposition in mixed hepatocytes/reticuloendothelial cells, and REC: iron deposition in reticuloendothelial cells alone.

Clinical and histological characteristics of the 48 patients who underwent phlebotomy are summarized in Table 4.  Figure 3(a) represents the change in ALT levels after phlebotomy. ALT levels were significantly decreased in the HC and Mix groups. The decrease in ALT levels in the REC group did not achieve statistical significance due to the small number of patients. The effects of phlebotomy on ALT levels tended to be greater in the Mix and REC groups than in the HC group (*P* = 0.082, ANOVA) (Figure 3(b)).

## 4. Discussion

The current study showed high frequency of stainable hepatic iron in patients with CHepC, as previously reported [1–3]. Overall, 61% of the patients had stainable iron either in HC, REC, or both cell types. HC iron scores were mild except in the 11% of patients who had severe HC iron scores. Although it was not examined whether the patients were genetically predisposed to iron overload, the previously reported prevalence of the mutations of the HFE gene in Japanese population is less than 1% [25].

First, relationships between hepatic iron distribution and biochemical and histological findings of CHepC were examined. It was found that REC iron depositions were significantly associated with the severities of liver function tests, stage, grade, and steatosis and were independently associated with advanced fibrosis. In contrast, HC iron itself seemed less significant than REC iron, because the liver function tests and scores of stage and grade were almost identical between patients with HC iron deposition alone and those without stainable iron. These findings expand Hézode's report that showed the association between liver cirrhosis and the presence of macrophage iron accumulation in CHepC [2]. The present study was unique for its examination of the levels of TNF-*α* and hepcidin mRNA. The cut-off value of ferritin for REC iron deposition was higher than that for HC iron deposition. Therefore, it can be assumed that iron deposition initially develops in HC followed by REC iron deposition in the development of CHepC.

The association between nonparenchymal iron deposition and disease severity has also been shown in patients with NASH and ALD [17, 18]. Taken together with the current study, it is likely that nonparenchymal iron deposition is a common feature of progressing chronic liver diseases.

Second, the pathogenesis of CHepC was examined in terms of TNF-*α* production. TNF-*α* has been implicated as an important pathogenic mediator in a variety of liver diseases [26]. Serum sTNFR2 levels, which have been shown to reflect disease progression in CHepC [27], were significantly higher in patients with REC iron than in those without REC iron. Moreover, REC iron scores were independently correlated with sTNFR2 levels. Thus, the increase in TNF-*α* production suggests that disease progression is closely associated with iron deposition in REC.

The progression of hepatic fibrosis is driven by activated hepatic stellate cells (HSC). Our findings indicated that iron-loading in nonparenchymal, not parenchymal, cells was correlated with progressive fibrosis in CHepC. Although oxidative stress has been shown to activate HSC [28], the effect of iron-induced oxidative stress on HSC may depend on the localized environment of iron-filled cells. Using a rodent model of secondary iron overload, iron deposition in nonparenchymal Kuppfer cells was shown to induce HSC proliferation and activation, leading to liver cirrhosis [29]. These phenomena, however, were ameliorated by treatment with antioxidant [29]. Thus, the redox-active properties of localized iron deposition may be greater in REC than in HC. Alternatively, iron loading in REC may alter their redox status affecting cytokine production by these cells [30].

Third, the mechanisms involved in iron deposition in HC and REC were examined. It has been reported that HCV infection causes hepatic iron overload by the downregulation of hepcidin synthesis [15]. The current study showed that hepatic hepcidin mRNA levels were significantly increased in patients with HC iron than in those without stainable iron. When normalized relative to serum ferritin concentrations, however, the difference in hepcidin mRNA levels between these two groups was not significant. Moreover, normalized hepcidin mRNA levels tended to be lower in the Mix and REC groups than in the None group. The current study cannot verify the appropriateness of hepcidin production against iron overload because of the lack of data from the patients without HCV infection. With regard to the response to iron overload of hepcidin synthesis, Fujita et al. also reported that relative hepatic hepcidin mRNA levels to serum ferritin levels were low in CHepC compared to the other chronic liver diseases [31]. Thus, alterations in hepcidin synthesis may have facilitated hepatic iron deposition, especially in the Mix and REC groups. Since oxidative stress can affect hepcidin synthesis in hepatocytes [15], exacerbated oxidative stress resulting from iron deposition in REC may have affected hepcidin synthesis in hepatocytes, resulting in further hepatic iron overload.

Hepatic macrophages and Kupffer cells can take in iron exclusively through phagocytosis of senescent erythrocytes and/or damaged hepatocytes. Then, iron can be recycled to the blood through the iron-exporter ferroportin [8]. Therefore, ferroportin levels can affect iron sequestration within hepatic macrophages and Kupffer cells. However, the differences in hepcidin mRNA levels among the groups of patients with stainable iron did not reach statistical significance, making it difficult to determine whether hepcidin alone could affect iron deposition patterns. Mechanisms other than hepcidin may therefore be responsible for iron deposition in hepatic macrophages and Kupffer cells.

Finally, the effects of phlebotomy on ALT levels were examined. To the best of our knowledge, the current study is the first to compare the efficacy of phlebotomy among patients with different iron deposition patterns. Interestingly, the effects of phlebotomy on ALT levels tended to be greater in patients with REC iron deposition. These findings indicate the importance of iron reduction in nonparenchymal cells for inhibition of disease progression.

## 5. Conclusion

In summary, REC iron deposition in CHepC was associated with disease severities and enhanced production of TNF-*α*. Although inappropriate hepatic synthesis of hepcidin can promote hepatic iron deposition, additional mechanisms should be considered to explain how iron deposition patterns develop. Phlebotomy should be especially considered for patients with nonparenchymal hepatic iron deposition.

## Figures and Tables

**Table 1:** Clinical characteristics of patients.

Group	None (*n* = 141)	HC (*n* = 107)	Mix (*n* = 101)	REC (*n* = 24)	ANOVA
Age	54.5 ± 12.3	55.9 ± 11.0	56.7 ± 11.0	59.1 ± 12.1	0.216
Gender (male/female)	37/104	52/55	68/33	14/10	<0.0000001*
BMI (kg/m^2^)	22.7 ± 4.0	22.8 ± 3.5	23.9 ± 3.4	23.7 ± 3.6	0.037
Diabetes (yes/no)	10/131	10/97	9/92	2/22	0.925*
Genotype (1a/lb/2a/2b/3a)	2/61/32/11/1	1/67/11/4/0	2/50/11/11/0	0/12/3/2/0	0.185*
Serogroup (G1/G2)	25/9	18/6	21/6	5/2	0.985*
HCV-RNA (logIU/mL)	5.9 ± 0.8	6.1 ± 0.8	6.0 ± 0.8	6.1 ± 0.8	0.057
AST (IU/L)	51.3 ± 36.5	43.9 ± 28.9	72.6 ± 39.1^cg^	81.9 ± 62.0^bf^	<0.0000001
ALT (IU/L)	59.6 ± 53.0	53.7 ± 35.2	101.2 ± 66.9^dg^	94.7 ± 75.5^ae^	<0.0000001
Triglyceride (mg/dL)	103.2 ± 86.8	100.9 ± 56.6	99.2 ± 42.0	103.5 ± 34.4	0.984
IRI (*μ*U/mL)	10.7 ± 11.7	8.3 ± 3.7	11.2 ± 5.7	11.5 ± 6.0	0.341
FPG (mg/dL)	99.9 ± 25.6	98.7 ± 13.4	102.6 ± 17.7	104.2 ± 26.0	0.468
HOMA-R	3.4 ± 6.3	2.1 ± 1.1	2.8 ± 1.4	3.0 ± 1.8	0.424
Ferritin (ng/mL)	93.9 ± 85.1	191.5 ± 109.1^b^	425.1 ± 300.9^dgh^	215.4 ± 155.6^a^	<0.0000001
Stage (0/1/2/3/4)	1/82/42/15/1	2/60/36/9/0	1/29/41/21/9	0/3/11/5/5	<0.0000001
Grade (0/1/2/3)	4/79/53/5	2/67/31/7	0/31/55/15	0/2/15/7	<0.0000001
HC iron score (1/2/3/4)	—	63/32/10/2	30/42/21/8	—	—
REC iron score (1/2)	—	—	2.2	3.8	—
Steatosis (0/1/2/3)	91/28/19/3	67/26/13/1	39/36/19/7	7/10/4/3	<0.0005

^a^ *P* < 0.05, ^b^ *P* < 0.005, ^c^ *P* < 0.0001, ^d^ *P* < 0.0000001 versus None; ^e^ *P* < 0.01, ^f^ *P* < 0.0001, ^g^ *P* < 0.0000001 versus HC; and ^h^ *P* < 0.00005 versus REC (Bonferroni test). *Chi-squared test.

None: no stainable iron, HC: hepatocytes, Mix: mixed hepatocytes/reticuloendothelial cells, REC: reticuloendothelial cells, BMI: body mass index, AST: aspartate aminotransferase, ALT: alanine aminotransferase, IRI: immunoreactive insulin, FPG: fasting plasma glucose, and HOMA-R: homeostasis model assessment ratio.

**Table 2:** Logistic regression analysis of factors associated with advanced stage.

	Stage 0–2	Stage 3–4	Univariate	Multivariate
Age	54.2 ± 12.1	59.3 ± 9.9	0.019	0.968
Gender (male/female)	136/172	35/30	0.099*	—
BMI (kg/m^2^)	22.4 ± 3.1	26.6 ± 5.0	<0.000005	0.450
Diabetes (yes/no)	20/288	11/54	0.009*	0.072
Genotype (1a/1b/2a/2b/3a)	3/159/46/26/1	2/31/11/2/0	0.350*	—
Serogroup (G1/G2)	56/17	13/6	0.469*	—
HCV-RNA (logIU/mL)	6.0 ± 0.8	6.3 ± 0.7	0.536	—
AST (IU/L)	48.4 ± 33.7	80.9 ± 40.9	<0.0000001	<0.05
ALT (IU/L)	64.9 ± 59.5	90.2 ± 52.7	<0.0000001	<0.05
IRI (*μ*U/mL)	9.0 ± 6.4	14.9 ± 10.6	0.008	0.151
FPG (mg/dL)	100.2 ± 26.4	110.2 ± 31.8	<0.0001	0.675
HOMA-R	2.5 ± 3.9	4.7 ± 6.4	0.040	0.324
Ferritin (ng/mL)	214.1 ± 235.3	221.2 ± 185.8	0.034	<0.05
Grade (0/1/2/3)	6/177/115/10	0/2/39/24	<0.0000001*	<0.005
Steatosis (0/1/2/3)	188/78/37/5	16/22/18/9	<0.0000001*	0.811
HC iron (0/1/2/3/4)	139/76/57/28/8	26/17/17/3/2	0.511*	—
REC iron (0/1/2)	223/60/25	25/28/12	<0.0000005*	<0.05

BMI: body mass index, AST: aspartate aminotransferase, ALT: alanine aminotransferase, IRI: immunoreactive insulin, FPG: fasting plasma glucose, HOMA-R: homeostasis model assessment ratio, HC: hepatocytes, and REC: reticuloendothelial cells.

Univariate: Student's *t*-test, Multivariate: logistic regression, and *Chi-squared test.

**Table 3:** Variables associated with sTNFR2 levels.

	Coefficient	Univariate	Regression
Age	0.271	<0.001	<0.01
AST	0.387	<0.000005	0.363
ALT	0.367	<0.000005	0.628
Ferritin	0.302	<0.0005	0.929
Stage	0.292	<0.0005	0.394
Grade	0.389	<0.000005	<0.05
Steatosis	0.169	<0.05	0.434
REC iron score	0.401	<0.0000005	<0.005

Univariate: Pearson's correlation coefficient, and Regression: regression analysis.

AST: aspartate aminotransferase, ALT: alanine aminotransferase, and REC: reticuloendothelial cells.

**Table 4:** Forty-eight patients who underwent phlebotomy.

Age	57.2 ± 10.6
Gender (male/female)	31/17
Ferritin (ng/mL)	438.3 ± 322.1
ALT (IU/L)	109.8 ± 65.8
Stage (0/1/2/3/4)	0/10/27/9/2
Grade (0/1/2/3)	0/17/26/5
Steatosis (0/1/2/3)	14/18/5/11
Hepatocyte iron score (0/1/2/3/4)	5/18/10/13/2
REC iron score (0/1/2)	12/28/8
Pattern	
HC alone	12
Mixed HC/REC	31
REC alone	5

ALT: alanine aminotransferase, HC: hepatocytes, and REC: reticuloendothelial cells.
